# Organ-Specific Responses to Chronic High-Fat Diets in Mice: Insights into Phospholipid Fatty Acid Distribution

**DOI:** 10.3390/nu17050821

**Published:** 2025-02-27

**Authors:** Aleksandra Nenadovic, Sanjin Kovacevic, Anica Stankovic, Tamara Popovic, Jasmina Debeljak Martacic, Slavica Rankovic, Silvio R. De Luka, Jelena Milasin, Jelena Nesovic Ostojic

**Affiliations:** 1 Department of Pathological Physiology, Faculty of Medicine, University of Belgrade, 11000 Belgrade, Serbia; aleksandra.nenadovic@med.bg.ac.rs (A.N.); sanjin.kovacevic@med.bg.ac.rs (S.K.); anica.stankovic123@gamil.com (A.S.); silvio.de-luka@med.bg.ac.rs (S.R.D.L.); 2 Centre of Excellence in Nutrition and Metabolism, Institute for Medical Research, University of Belgrade, 11000 Belgrade, Serbia; poptam@gmail.com (T.P.); minaizdravko@yahoo.com (J.D.M.); vica0282@gmail.com (S.R.); 3 Department of Human Genetics, Faculty of Dental Medicine, University of Belgrade, 11000 Belgrade, Serbia; jelena.milasin@stomf.bg.ac.rs

**Keywords:** chronic high-fat diet, linseed oil, palm oil, sunflower oil, phospholipid fatty acid, organ-specific response, lipid profile, cardiovascular indices

## Abstract

**Background/Objectives**: This research aimed to investigate phospholipid fatty acid (PLFA) distribution in the brain, kidneys, and white adipose tissue (WAT) and lipid profiles in response to high-fat diets. **Methods**: Adult female C57BL/6 mice were fed high-fat diets containing 25% linseed, palm, or sunflower oil for 100 days. The fatty acid composition of dietary oils and tissue PL were analyzed using gas–liquid chromatography. **Results**: Linseed oil increased *n*-3 polyunsaturated fatty acids (PUFAs) with subsequent conversion into long-chain *n*-3 PUFAs in the brain and kidney PL, while only alpha-linolenic acid was elevated in WAT. Palm and sunflower oils resulted in unique PLFA distributions in the kidneys and WAT. Palm oil raised linoleic acid without conversion to pro-inflammatory *n*-6 PUFAs. Sunflower oil increased saturated palmitic acid, as opposed to the rise in monounsaturated oleic acid. Linseed oil also significantly improved lipid profiles, reducing LDL and increasing HDL levels while enhancing cardiovascular indices. **Conclusions**: This study demonstrates that dietary oils significantly impact organ-specific PLFA profiles, with linseed oil enriching brain and renal *n*-3 PUFAs, while palm and sunflower oils induce distinct modifications in the kidney and WAT. Moreover, linseed oil offers notable cardioprotective benefits due to the favorable lipid profile changes. These findings highlight the importance of dietary fat selection in achieving balanced lipid metabolism and suggest that diverse oil combinations may be essential for optimizing health outcomes.

## 1. Introduction

Contemporary lifestyles and dietary habits contribute to increased fat and caloric intake, leading to the pandemic of chronic non-infectious diseases [1]. Vegetable oils are an integral part of the human diet worldwide, providing energy and essential fatty acids (EFAs). EFAs include linoleic acid (LA) (18:2, *n*-6) and alpha-linolenic acid (ALA) (18:3, *n*-3), which must be obtained from food as the body cannot synthesize them. All other long-chain polyunsaturated fatty acids (PUFAs) are derived from LA and ALA [2]. Linseed oil is particularly rich in ALA, which, through metabolic processes, is converted into eicosapentaenoic acid (EPA) (20:5, *n*-3) and docosahexaenoic acid (DHA) (22:6, *n*-3) [3]. Palm oil mainly consists of saturated fatty acids (SFAs), such as palmitic acid (PA) (16:0) and stearic acid (STA) (18:0), along with monounsaturated fatty acids (MUFAs) and a small amount of PUFAs [4]. In contrast, sunflower oil is predominantly composed of unsaturated fatty acids like LA and oleic acid (OA) (18:1, *n*-9), with PA and STA present in low percentages [5].

Phospholipids (PLs) are composed of a phosphate group, a glycerol backbone, and two fatty acid chains. These amphipathic molecules are essential for forming the bilayer membrane of all cells [6]. The type of fatty acids in the PL composition significantly influences the membrane’s physical and chemical properties. SFAs increase the rigidity of the cell membrane, whereas MUFAs and PUFAs enhance membrane fluidity, thereby contributing to the cell’s thermodynamic stability [7]. Beyond their structural role, PLs also function as receptors, enzymes, ion channels, and precursors of secondary messengers, among other functions. Alterations in the fatty acyl chain composition of PL impact the biophysical characteristics of cell membranes, subsequently affecting their function [8]. Since the composition of PL is closely tied to dietary intake, consuming the right types of fatty acids is crucial for maintaining membrane stability and ensuring optimal organ function.

Modern dietary patterns dominate with SFAs, such as PA and STA. In addition to SFA, *n*-6 PUFAs are highly prevalent [9]. Arachidonic acid (AA) (20:4, *n*-6), a type of *n*-6 PUFA, has a pro-inflammatory potential, and its increased incorporation into the PL contributes to enzyme conversion to inflammatory eicosanoids [10]. In contrast, *n*-3 PUFAs are found in much lower concentrations in food. Incorporating long-chain *n*-3 PUFAs into PL exerts anti-inflammatory effects, reducing leukocyte chemotaxis, lowering free radical production, and decreasing levels of pro-inflammatory mediators [11]. Due to these beneficial effects of *n*-3 PUFAs on the body, an increased intake is recommended while maintaining an optimal *n*-6/*n*-3 PUFA ratio, ranging from 1:1 to 1:5 [12].

Previous studies have shown that the composition of phospholipid fatty acids (PLFAs) varies significantly among different organs and that dietary intake plays a key role in influencing these organ-specific alterations [13,14]. Dietary fats are crucial in modulating brain functions by influencing the composition of neuronal membranes and the balance of neuroactive lipids [15]. High-fat diets, depending on their fatty acid composition, can significantly alter brain PLFAs, impacting synaptic plasticity, neurotransmission, and neuroinflammation [16]. For instance, diets high in saturated fats have been linked to harmful effects on cognitive functions and increased inflammation [17], while those rich in PUFAs, such as *n*-3 fatty acids, are associated with neuroprotective effects [18]. On the other hand, the kidneys are crucial in maintaining homeostasis, including lipid metabolism and excretion [19]. Chronic high-fat diets have been shown to induce significant alterations in the renal PLFA profile, which may contribute to kidney dysfunction and the development of metabolic disorders [20]. The specific composition of dietary fats influences the incorporation of fatty acids into renal PLs, thereby affecting membrane fluidity, signaling pathways, and renal cell function. These alterations could potentially exacerbate oxidative stress and inflammation, processes known to be involved in the progression of chronic kidney disease (CKD) and other renal pathologies [21]. In addition, white adipose tissue (WAT) plays a complex role in regulating systemic metabolism, serving as a primary energy reservoir and an active endocrine organ [22]. In the modern lifestyle, high-fat diets, typically rich in SFA and *n*-6 PUFA, contribute to an imbalance between caloric intake and expenditure, promoting adipose tissue expansion and remodeling. This expansion, mainly in visceral depots, is strongly associated with metabolic disorders, as visceral obesity is linked to increased inflammation and a higher risk of chronic metabolic diseases [23,24].

In our previous research, we investigated PLFA distribution in the liver—a central organ for lipid metabolism—in response to chronic high-fat diets rich in linseed, palm, and sunflower oils [25]. Building on these findings, we expanded our research to focus on the brain, kidneys, and WAT. These organs are crucial for understanding the systemic impact of dietary fats, as they differ in their metabolic roles and sensitivity to lipid-induced changes. Therefore, this study aimed to simultaneously examine how the chronic consumption of linseed, palm, or sunflower oil affects PLFA composition in these tissues using the well-established C57BL/6 mouse model for lipid metabolism research [26,27]. The 14-week high-fat diet regimen was chosen to capture metabolic adaptations and lipid profile changes simulating chronic dietary exposure. Importantly, this period corresponds to approximately ten years in human terms [28], underscoring the translational relevance of our findings. Our research provides valuable insights into how the long-term consumption of specific dietary oils can differentially influence organ-specific PLFA distribution, informing potential nutritional strategies for promoting human health.

## 2. Materials and Methods

### 2.1. Experimental Animals

A total of 24 adult female C57BL/6 mice, obtained from the Galenika vivarium (Galenika a.d., Belgrade, Serbia), 6 weeks old and having about 20 g weight were used in this study. The mice were randomly assigned to four equal groups, with six animals in each group. The sample size calculation for the number of experimental units per group and the total number of animals was calculated using the appropriate formula [29]. Mice were housed at three animals per cage, obtained randomly from the pool of all cages, under identical and controlled conditions, with a room temperature of 22 ± 1 °C, humidity of 65 ± 1%, and a 12 h light/dark cycle. Animals were monitored twice daily, and their health status was estimated by a general assessment of animal activity, food and water intake, external appearance, and absence of disease. All experimental procedures were reviewed and approved by the Experimental Animals Ethics Committee at the University of Belgrade, Faculty of Medicine (approval number 323-07-09403/2014-05/1, dated 13 November 2015). The study adhered to the ARRIVE guidelines and the National Research Council’s Guide for the Care and Use of Laboratory Animals.

### 2.2. Chronic High-Fat Diets

This study included mice on different dietary regimens for 14 weeks, with free access to feed and drinking water throughout the study. Before the experiment, 1 week of acclimatization was performed in order to prevent the influence of stress on the experimental outcome. The control group consumed the standard mouse chow sourced from the Veterinary Institute Subotica (Subotica, Serbia), which served as the baseline diet. In contrast, the remaining three groups received high-fat diets that were isocaloric with the control diet but enriched with 25% of either linseed, palm, or sunflower oil (GA-ME-HA, Sarajevo, Bosnia and Herzegovina). This dietary composition was intended to simulate chronic high-fat conditions. The detailed composition of the standard mouse chow and fatty acid profiles of the linseed, palm, and sunflower oils were established and reported in our previous study [25]. The performed experimental protocol did not cause pain, suffering, and distress to the experimental animals or any unexpected adverse events.

### 2.3. Organ Sample Collection and Preparation

After the 14-week dietary regimen, 24 mice without exclusion, previously anesthetized, were euthanized with cervical dislocation. The blood samples were collected and centrifuged to separate serum from erythrocytes. Serum samples were stored at −20 °C until analysis. Further dissection was performed, and the endocranium and abdominal cavity were surgically opened to collect brain, kidney, and visceral white adipose tissue samples. Each organ was carefully extracted, and approximately 1 g of tissue from each organ was immediately frozen at –80 °C to preserve the integrity of the samples. Subsequently, the frozen brain, kidney, and white adipose tissue samples were homogenized using an ultrasonic homogenizer (Bandelin Sonoplus, Bandelin electronic, Berlin, Germany) to prepare them for fatty acid analysis. This process ensured that the tissue samples were adequately prepared for subsequent biochemical assessments. All further analyses were performed by investigators that were blinded to the experimental protocol.

### 2.4. Analysis of Phospholipid Fatty Acids in Brain, Kidney, and White Adipose Tissue

Brain, kidney, and white adipose tissue samples were prepared for fatty acid analysis by focusing on their PL content. Approximately 1 g of each tissue was lyophilized to remove moisture. After lyophilization, the dried tissue was manually pulverized using a mortar and pestle to increase the surface area, thus enhancing lipid extraction efficiency. The pulverized tissue was then subjected to lipid extraction using a chloroform/methanol (2:1) mixture, which included butylated hydroxytoluene (BHT) as an antioxidant to prevent oxidation. The lipid extract was washed with 0.2 volumes of water relative to the total volume of the chloroform/methanol mixture used. Specifically, for every 1 mL of chloroform/methanol extract, 0.2 mL of water was added. The solution was then centrifuged, and the upper aqueous phase was discarded.

Further purification was achieved through successive solvent evaporations. The solvents used included methanol/benzene (2:1), acetone/benzene (2:1), and ethanol/benzene (2:1). Following these steps, chloroform was added to the remaining extract and evaporated, and the sample was subsequently prepared by adding hexane. This preparation was essential for the next step, which involved thin-layer chromatography (TLC).

PLs were isolated from the lipid extract using one-dimensional TLC with a neutral lipid solvent system comprising hexane-diethyl ether-acetic acid (87:2:1, *v*/*v*/*v*) on Silica Gel GF plates (C. Merck, Darmstadt, Germany). For the fatty acid analysis, fatty acid methyl esters were prepared and analyzed via gas–liquid chromatography (GLC) following established protocols [30]. Individual fatty acid methyl esters were identified by comparing their retention times with those of authentic standards (Sigma Chemical Co., St. Louis, MO, USA) and/or the PUFA-2 standard mixture (Supelco Inc., Bellefonte, PA, USA) [25].

### 2.5. Estimation of Enzyme System Activities

The activities of key enzymes involved in fatty acid metabolism were estimated based on the ratios of product-to-precursor fatty acids. Specifically:

1. Elongase Activity (EA): $EA=\frac{STA}{PA}$

where: STA = stearic acid (18:0); PA = palmitic acid (16:0).

2. Δ9 Desaturase Activity (D9D): $D9D=\frac{OA}{STA}$

where: OA = oleic acid (18:1, *n*-9); STA = stearic acid (18:0).

3. Δ6 Desaturase Activity (D6D): $D6D=\frac{GLA}{LA}$

where: GLA = gamma-linolenic acid (18:3, *n*-6); LA = linoleic acid (18:2, *n*-6).

4. Δ5 Desaturase Activity (D5D): $D5D=\frac{AA}{DGLA}$

where: AA = arachidonic acid (20:4, *n*-6); DGLA = dihomo-gamma-linolenic acid (20:3, *n*-6).

This ratio-based method for estimating elongase and desaturase enzyme activities is well-established and widely used in lipid research [31,32].

### 2.6. Analysis of Serum Lipid Profile, Cardiovascular Indices, and C-Reactive Protein

The concentrations of total cholesterol (TC), high-density lipoprotein cholesterol (HDL), low-density lipoprotein cholesterol (LDL), triglycerides (TGs), and C-reactive protein (CRP) were measured using the COBAS INTEGRA 400 plus automated analyzer (Hoffmann-La Roche, Mannheim, Germany).

The concentrations of TC, TG, HDL cholesterol, and LDL cholesterol (all in mmol/L) were used to calculate several atherogenic and cardioprotective indices. The formulas applied were as follows [33]:

1. Atherogenic Index (AI): $\mathrm{AI}=\log(\frac{\mathrm{TG}}{\mathrm{HDL}})$,

2. Atherogenic Coefficient (AC): $\mathrm{AC}=\frac{\mathrm{TC}-\mathrm{HDL}}{\mathrm{HDL}}$,

3. Cardiac Risk Ratio (CRR): $\mathrm{CRR}=\frac{\mathrm{TC}}{\mathrm{HDL}}$,

4. Cardioprotective Index (CPI): $\mathrm{CPI}=\frac{\mathrm{HDL}}{\mathrm{LDL}}$.

These indices were analyzed to evaluate the impact of dietary interventions on lipid metabolism and cardiovascular risk markers.

Additionally, non-HDL cholesterol (non-HDL-c) was calculated using the formula:

non-HDL-c = TC – HDL

### 2.7. Data and Statistical Evaluation

Statistical assessment was performed using GraphPad Prism software, version 10.2.3 (347) (GraphPad Software, Boston, MA, USA). The data assumptions were assessed using Levene’s Test for homogeneity of variance. To compare data across different groups, one-way ANOVA was conducted, followed by Tukey’s post hoc test for pairwise comparisons. The statistical analysis excluded any values that were not detected. The results are expressed as mean ± standard deviation (SD). Statistical significance was determined at thresholds of *p* ≤ 0.05 for significance and *p* ≤ 0.01 or *p* ≤ 0.001 for high significance.

Correlation analysis was employed using Pearson’s correlation for variables with homogeneous distributions and Spearman’s rank correlation for non-homogeneous distributions. Data are presented as correlation coefficients (r) and corresponding *p*-values. Statistical significance was considered relevant for *p* < 0.05.

## 3. Results

### 3.1. Brain Phospholipid Fatty Acid Distributions

In the brain PL, STA concentration was the lowest after the linseed oil diet compared to all other groups. LA levels were elevated in the brain PL after the linseed and palm oil diets compared to the control and sunflower oil groups. Additionally, the percentage of DGLA was the highest in the brain PL after the linseed oil treatment compared to the other three groups. The linseed oil diet also increased the EPA concentration in the brain PL compared to all other groups. Moreover, the linseed oil elevated the docosapentaenoic acid (DPA) (22:5, *n*-3) percentage in the brain PL compared to the control and palm oil diets (refer to Table 1).

### 3.2. Brain Enzyme Activity Estimations

**Figure 1:**
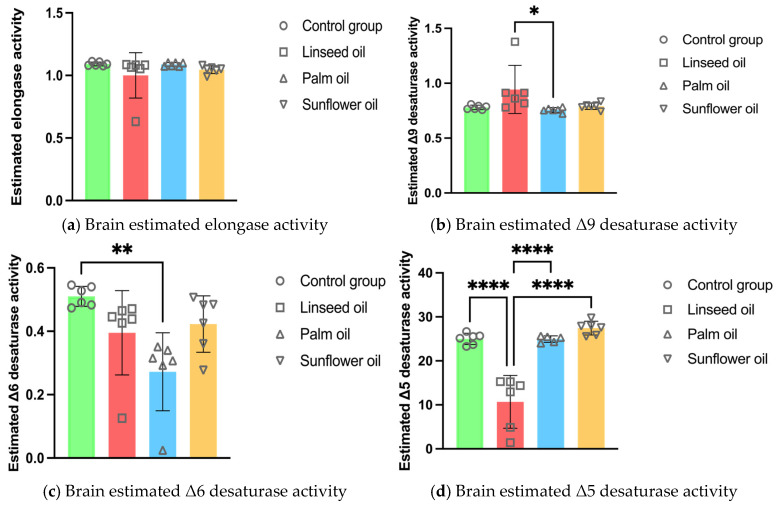
Brain enzyme activity estimations in mice fed regular chow (control group), linseed oil, palm oil, and sunflower oil diets: (**a**) brain estimated elongase activity (n = 6); (**b**) brain estimated Δ9 desaturase activity (n = 6); (**c**) brain estimated Δ6 desaturase activity (n = 6); (**d**) brain estimated Δ5 desaturase activity (n = 6, except n = 5 in the palm oil group). Data are given as mean ± standard deviation (SD). One-way ANOVA followed by Tukey’s post hoc test was conducted for pairwise comparisons between all groups. The statistical analysis did not include the undetected values. Statistical significance: * *p* < 0.05; ** *p* < 0.01; **** *p* < 0.0001.

The estimated activity of Δ9 desaturase in the brain was higher after linseed oil treatment compared to the palm oil diet. In contrast, the palm oil diet decreased the activity of Δ6 desaturase in the brain compared to the control group. Furthermore, the Δ5 desaturase activity in the brain was the lowest after the linseed oil diet compared to all other groups (shown in Figure 1).

### 3.3. Kidney Phospholipid Fatty Acid Distributions

In the kidney PL, the total SFA percentage decreased after linseed and palm oil diets compared to the control group. Also, the linseed and palm oil lowered the PA concentration in the renal PL compared to the control and sunflower oil groups. STA levels in the kidney PL were the lowest after sunflower oil treatment compared to the other three groups.

The total MUFA percentage in the kidney PL was the highest after the sunflower oil diet compared to all other groups. The palmitoleic acid (POA) (16:1, *n*-7) levels were higher after the sunflower oil diet compared to the palm oil treatment. However, POA levels were lower after the linseed and palm oil diets compared to the control group. Moreover, the sunflower oil elevated the OA concentration in the renal PL compared to all other diets. All high-fat diets lowered the vaccenic acid (VA) (18:1, *n*-7) levels in the kidney PL compared to the control group.

The total PUFA rate in the kidney PL increased after the linseed and palm oil compared to the control and sunflower oil diets. The overall percentage of *n*-6 PUFAs in the renal PL was the highest after the palm oil treatment compared to all other groups. Yet, the sunflower oil diet lowered the overall *n*-6 PUFAs compared to the control group. Levels of a single *n*-6 PUFA, LA, were elevated after the palm oil diet compared to all other groups. Furthermore, the sunflower oil treatment decreased the LA and GLA concentrations in the kidney PL compared to the linseed oil diet. However, the linseed oil diet significantly elevated the total *n*-3 PUFAs and the levels of individual *n*-3 PUFAs, including ALA, EPA, and DPA, in the kidney PL compared to the other three groups.

The *n*-6/*n*-3 ratio was the lowest after the linseed oil diet and the highest after the palm oil diet compared to all other groups. Additionally, the linseed oil diet increased the EPA/AA ratio in the kidney PL compared to the other three diets (check Table 2 for details).

### 3.4. Kidney Enzyme Activity Estimations

**Figure 2:**
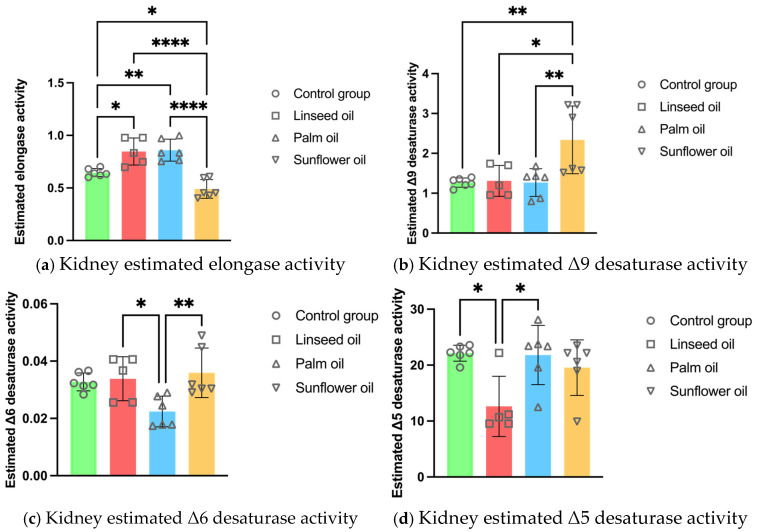
Kidney enzyme activity estimations in mice fed regular chow (control group), linseed oil, palm oil, and sunflower oil diets: (**a**) kidney estimated elongase activity (n = 6, except n = 5 in the linseed oil group); (**b**) kidney estimated Δ9 desaturase activity (n = 6, except n = 5 in the linseed oil group); (**c**) kidney estimated Δ6 desaturase activity (n = 6, except n = 5 in the linseed oil group); (**d**) kidney estimated Δ5 desaturase activity (n = 6, except n = 5 in the linseed oil group). Data are given as mean ± standard deviation (SD). One-way ANOVA followed by Tukey’s post hoc test was conducted for pairwise comparisons between all groups. The statistical analysis did not include the undetected values. Statistical significance: * *p* < 0.05; ** *p* < 0.01; **** *p* < 0.0001.

The estimated elongase activity in the kidney PL increased after the linseed and palm oil diets compared to the control group. The sunflower oil diet decreased the elongase activity while elevating the Δ9 desaturase activity in the kidneys compared to the other three treatments. Moreover, the Δ6 desaturase was less active in the renal PL after the palm oil diet compared to the other high-fat diets. The linseed oil treatment lowered the Δ5 desaturase activity in the kidney PL compared to the control and sunflower oil groups (see Figure 2).

### 3.5. White Adipose Tissue Phospholipid Fatty Acid Distributions

The sunflower oil diet increased the total SFA and individual PA in the adipocyte PL compared to the linseed and palm oil diets. Furthermore, PA levels decreased after the linseed and palm oil treatments compared to the control group.

The total MUFA and individual OA percentage in the adipocyte PL increased after sunflower oil consumption compared to all other groups. Moreover, the POA concentration in the adipocyte PL decreased after the high-fat treatments compared to the control group. However, the POA concentration was higher after the sunflower oil diet compared to the palm oil diet. The VA levels decreased in the adipocyte PL after the high-fat diets compared to the control group, with the most prominent decrease observed after the palm oil treatment.

The total PUFA percentage in the adipocyte PL was the lowest after the sunflower oil diet compared to all other groups but higher after the palm oil diet compared to the control group. Also, the palm oil treatment elevated the overall *n*-6 PUFAs and individual LA compared to the other three diets. In contrast, the sunflower oil diet decreased the *n*-6 PUFAs and LA compared to the control group. Meanwhile, the GLA concentration was significantly increased after the linseed oil consumption in the adipocyte PL compared to the other three diets. However, the AA levels in the adipocyte PL were reduced after the linseed oil diet compared to the palm oil diet. The linseed oil diet dominantly elevated total *n*-3 PUFAs and individual ALA in the white adipose tissue PL compared to all other groups.

The *n*-6/*n*-3 ratio was the lowest after the linseed oil diet and the highest following the palm oil diet compared to all other groups. Similarly, the EPA/AA ratio was the highest after the linseed oil treatment compared to all other groups. The unsaturation index (UI) in the adipocyte PL was reduced following the consumption of sunflower oil compared to other high-fat diets. Nevertheless, the UI increased after the linseed and palm oil treatments compared to the control group (refer to the data in Table 3).

### 3.6. White Adipose Tissue Enzyme Activity Estimations

**Figure 3:**
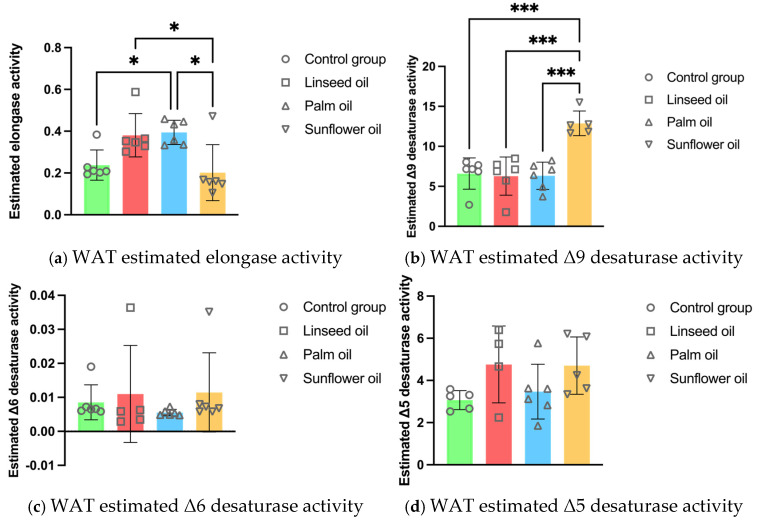
White adipose tissue (WAT) enzyme activity estimations in mice fed regular chow (control group), linseed oil, palm oil, and sunflower oil diets: (**a**) WAT estimated elongase activity (n = 6); (**b**) WAT estimated Δ9 desaturase activity (n = 6, except n = 5 in the sunflower oil group); (**c**) WAT estimated Δ6 desaturase activity (n = 6, except n = 5 in the linseed oil group); (**d**) WAT estimated Δ5 desaturase activity (n = 6, except n = 5 in the control and sunflower oil groups, n = 4 in the linseed oil group). Data are given as mean ± standard deviation (SD). One-way ANOVA followed by Tukey’s post hoc test was conducted for pairwise comparisons between all groups. The statistical analysis did not include the undetected values. Statistical significance: * *p* < 0.05; *** *p* < 0.001.

The estimated elongase activity in the adipocyte PL was lower after the sunflower oil consumption compared to the linseed and palm oil diets. However, the calculated elongase activity was higher after the palm oil diet compared to the control group. The sunflower oil diet significantly increased the Δ9 desaturase activity in the white adipose tissue PL compared to all other treatments (illustrated in Figure 3).

### 3.7. Serum Lipid Profile, Cardiovascular Indices, and C-Reactive Protein

The lipid profile changes were observed dominantly after chronic linseed oil consumption. Specifically, the linseed oil significantly decreased LDL levels compared to all other treatments while elevating HDL concentration compared to the control group. Moreover, AC and CRR decreased after the linseed oil diet compared to the control and sunflower oil groups, but surprisingly, it was not the case compared to the palm oil diet. Notably, CPI was the highest in the linseed oil group compared to all other dietary groups (refer to Table 4 for details).

**Figure 4:**
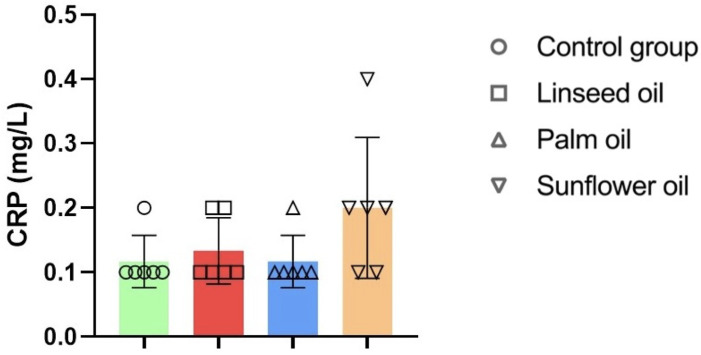
C-reactive protein (CRP) analysis in mice fed regular chow (control group), linseed oil, palm oil, and sunflower oil diets; n = 6 for all groups. One-way ANOVA followed by Tukey’s post hoc test was conducted for pairwise comparisons between all groups. Data are given as mean ± standard deviation (SD). The detection limit for CRP was 0.2 mg/L, and values below this threshold are presented as 0.1 mg/L in the figure.

However, there were no statistically significant differences in CRP levels across the dietary groups (shown in Figure 4).

### 3.8. Serum Lipid Correlations with Fatty Acid Distribution

Correlation analysis identified significant relationships between lipid levels and fatty acid distribution across different organs. TC negatively correlated with SFA, PA, LA, *n*-3 FA, DHA, and the EPA/AA ratio in the brain PL of mice fed palm oil, as well as with DGLA and DPA in the kidney PL of mice fed linseed oil and DGLA in the palm oil group (Supplementary Table S1). LDL exhibited a positive correlation with ALA in the brain PL of linseed oil-fed mice, while a negative correlation with SFA in the palm oil group. In white adipose tissue, LDL was positively correlated with *n*-6 FA and EPA/AA in the linseed oil group and with *n*-6 FA, AA, DHA, and UI in the sunflower oil group (Supplementary Table S2). HDL was negatively correlated with SFA, LA, *n*-3, DHA, and the EPA/AA ratio in the brain PL of palm oil-fed mice. In the kidney PL, HDL was negatively correlated with LA but positively correlated with DHA in the linseed oil group, while in the palm oil group, it showed a negative correlation with DGLA (Supplementary Table S3). Lastly, TG was negatively correlated with SFA, PA, POA, *n*-3, and DHA in the brain PL of palm oil-fed mice and with DGLA in the kidney PL of linseed oil-fed mice (Supplementary Table S4).

## 4. Discussion

Dietary habits impact the composition of PLFAs and are critical for maintaining organ-specific functions, as these fatty acids play a pivotal role in stabilizing cell membranes [34,35]. In this study, we demonstrated that chronic high-fat diets rich in linseed, palm, or sunflower oil significantly influence the incorporation of fatty acids into the PL membranes of various organs. Our findings reveal that the type of dietary fat consumed not only determines the PLFA profile but also affects this profile in an organ-specific manner. Previously, we examined the liver’s response to these high-fat diets as a central organ for lipid metabolism, showing distinct changes in PLFA distribution [25]. Extending this research, we now report that similar dietary interventions result in differential PLFA compositions in the brain, kidneys, and white adipose tissue, underscoring the unique metabolic responses of each organ to chronic high-fat intake.

The type of fat consumed influences the incorporation of dietary fatty acids into brain PLs, with significant variations observed in response to linseed, palm, or sunflower oil. Our findings reveal significant alterations in the brain PLFA composition, with the linseed oil diet leading to the most prominent changes. This effect is likely due to the high content of *n*-3 ALA in linseed oil [25], which has been shown to profoundly influence the brain’s fatty acid profile [36]. Notably, STA concentrations were the lowest following the linseed oil diet, reflecting reduced incorporation of SFAs into brain PL. SFAs have been shown to increase inflammation in both microglia and neurons, which are critical for maintaining learning and memory functions [37]. The elevated levels of LA and DGLA in the linseed oil group further highlight the diet’s impact on *n*-6 PUFA metabolism. The increase in EPA and DPA also underscores linseed oil’s potent *n*-3 enriching effect, promoting neuroprotective outcomes. These long-chain *n*-3 fatty acids enhance neuronal function and survival by supporting membrane fluidity and facilitating efficient neurotransmission [38]. Their anti-inflammatory properties further contribute to neuroprotection by reducing oxidative stress and protecting against neuronal damage [39,40].

The observed changes in estimated enzyme activities are particularly noteworthy. The elevated Δ9 desaturase activity following the linseed oil diet suggests an enhanced conversion of STA to OA, which may contribute to maintaining membrane fluidity [41]. Conversely, the decreased Δ5 desaturase activity, which was the lowest in the linseed oil group, indicates a metabolic shift where *n*-6 PUFA metabolism halts at DGLA. This finding is crucial, as DGLA is known to have anti-inflammatory and anti-proliferative properties [42], contrasting with AA, which is a precursor to pro-inflammatory eicosanoids [10]. The lack of conversion to AA in the linseed oil group suggests a potentially protective mechanism against neuroinflammation. Moreover, DGLA itself has been shown to have direct effects on cell membranes, helping stabilize them and protect against oxidative stress, which further contributes to its neuroprotective properties [43].

In this study, high-fat diets rich in linseed, palm, or sunflower oil resulted in distinct changes in renal PLFA composition. Chronic sunflower oil consumption resulted in a distinct renal PLFA profile, characterized by a significant elevation in saturated PA compared to palm and linseed oils, while STA concentration was the lowest across all groups. This suggests an insufficient elongation of PA into STA by the lowest estimated elongase activity observed after sunflower oil consumption. The observed elevation in PA after the sunflower oil diet raises some concerns, as excess PA has been shown in previous studies to induce apoptosis in kidneys [44]. Despite these changes, sunflower oil increased total MUFAs associated with various health benefits [45]. This rise is primarily attributed to the elevated incorporation of OA into renal PL. Additionally, sunflower oil induced the highest Δ9 desaturase activity, an enzyme responsible for converting STA into OA by introducing a double bond. This elevated activity might also explain the reduced STA levels in renal PL after sunflower oil consumption, as more STA is converted into OA. Our findings indicate that the sunflower oil diet has complex and controversial effects on renal PLFA profile, with both potentially harmful and beneficial outcomes.

Long-term linseed and palm oil diets significantly elevated the total PUFAs in renal PL compared to the control and sunflower oil groups. Palm oil specifically increased the total *n*-6 PUFAs, with a marked insertion of LA, leading to the highest *n*-6/*n*-3 ratio. Despite the high *n*-6 PUFA levels, there was no further conversion of LA into AA, associated with proinflammatory potential [10]. This suggests that chronic palm oil consumption may not induce unfavorable lipid metabolism in the kidneys, although further research is needed to clarify its long-term effects. On the other hand, the linseed oil diet significantly elevated the total *n*-3 PUFAs in renal PL, with higher incorporation of ALA, EPA, and DPA compared to all other treatments. The increased levels of *n*-3 PUFAs are known for their anti-inflammatory and protective roles in kidneys, especially in acute kidney injury (AKI) models [46,47,48]. Long-chain *n*-3 PUFAs, such as EPA, DPA, and DHA, compete with *n*-6 AA for the same enzymes for conversion into eicosanoid mediators with anti-inflammatory properties [49]. As expected, the *n*-6/*n*-3 ratio was the lowest after linseed oil consumption, emphasizing the beneficial shift towards a more favorable *n*-3 PUFA profile. However, it is important to acknowledge that improved renal PLFA profiles do not always translate into better kidney function, particularly in pathological conditions. For instance, a study on rats showed that despite modifying lipid mediator profiles, *n*-3 PUFA supplementation did not affect renal insufficiency or protect against damage in ischemia-reperfusion injury (IRI) models [50]. Similarly, another study on mice indicated that while *n*-3 PUFAs improved tubular function in AKI, they did not improve renal function or reduce inflammation [51]. Thus, although *n*-3 PUFAs exhibit promising effects on renal lipid metabolism, further research is needed to fully understand their limits and therapeutic potential in pathological conditions such as AKI and IRI.

Dietary fats significantly influence the fatty acid composition of adipocyte PL, impacting the pro- or anti-inflammatory profile of WAT [52]. While previous studies have predominantly focused on TG, our research provides novel insights into non-TG lipids, particularly the PLFA composition of visceral WAT, highlighting the distinct effects of chronic high-fat diets enriched with linseed, palm, or sunflower oil. Our results demonstrate that chronic sunflower oil consumption significantly increased total SFA in the PL membrane of visceral adipocytes, with a significant elevation in PA compared to linseed and palm oil diets. This increase in PA was accompanied by the lowest estimated elongase activity, which is responsible for converting PA into STA, highlighting a distinct metabolic response in WAT following sunflower oil intake. The elevated SFA content in adipocyte PLs is of particular concern, as SFAs increase membrane rigidity, potentially impairing insulin sensitivity and promoting the development of metabolic disorders [53,54]. However, recent findings challenge this notion, showing that elevated SFA levels in adipocyte PL do not necessarily impair insulin sensitivity, as demonstrated in vitro in human preadipocytes and in vivo in mouse WAT [55]. Furthermore, the sunflower oil diet increased the total MUFA in WAT PL, particularly OA, compared to all other groups. MUFAs, especially OA, are known to exert anti-inflammatory effects, counteracting the harmful impact of SFAs on adipose tissue function [56]. This rise in MUFA was associated with the highest Δ9 desaturase activity, which converts saturated STA into monounsaturated OA. The Δ9 desaturase activity has been shown to moderate the adverse effects of SFAs like PA and STA on cellular stress and cytokine production [57]. Despite the increase in SFAs, the rise in MUFAs, particularly OA, indicates that sunflower oil may balance metabolic risks by enhancing membrane fluidity and reducing inflammation in WAT.

Changes in the PUFAs in the visceral adipocyte PL followed expected patterns, with the long-term palm oil diet elevating total *n*-6 PUFA due to LA increase compared to all other groups. Additionally, palm oil increased AA percentage in WAT PL compared solely to the linseed oil diet. Palm oil consumption is associated with inflammation and dysregulated lipid metabolism in visceral WAT [58]. Despite palm oil’s high PA and total SFA content, these were unexpectedly lower in adipocyte PL compared to the control group. In the study on male rats treated with palm oil for 15 weeks, the PLFA profile in visceral fat showed the opposite trend, with high PA and low LA levels in WAT [59]. The opposing PLFA profile in visceral adipocytes may be influenced by species and sex differences, with a higher visceral fat distribution [60] and higher metabolic rates of long-chain fatty acids in female mice [61] and humans [62]. Conversely, chronic linseed oil consumption significantly increased total *n*-3 PUFA, particularly ALA in adipocyte PL membrane, compared to all other treatments. However, no further elongation of essential *n*-6 LA and *n*-3 ALA was observed in the visceral WAT. Linseed oil, rich in *n*-3 ALA, sustains beneficial adipocyte metabolism and reduces inflammatory mediator synthesis [63,64]. Our results demonstrated a favorable PLFA profile in WAT in response to the linseed oil, with the lowest *n*-6/*n*-3 ratio among all treatments. Furthermore, the EPA/AA ratio, a marker of inflammatory potential, was elevated following the linseed oil diet compared to the sunflower oil and control groups.

The findings of this study highlight the significant impact of vegetable oils on the serum lipid profile and their potential implications for cardiovascular health. Chronic consumption of linseed oil demonstrated the most pronounced cardioprotective effects, evidenced by decreased LDL levels, increased HDL concentrations, and reductions in the AC and CRR. The highest CPI observed in the linseed oil group further supports its favorable lipid-modulating properties. These results align with previous studies demonstrating the beneficial effects of *n*-3 polyunsaturated fatty acids in reducing cardiovascular risk markers [65,66,67]. Interestingly, while AC and CRR were lower in the linseed oil group compared to the control and sunflower oil groups, no such differences were noted compared to the palm oil group, suggesting a unique lipid profile response to palm oil consumption that warrants further investigation [68]. The absence of statistically significant differences in CRP levels across groups indicates that the observed lipid profile changes were not accompanied by marked systemic inflammation. However, multiple factors could influence CRP levels, so the lipid-centric effects of these dietary interventions should be interpreted while considering other potential inflammatory mechanisms.

The observed correlations between serum lipids and PLFA distribution do not follow a clear or expected pattern, necessitating cautious interpretation. Notably, all serum lipids (TC, LDL, HDL, and TG) consistently showed negative correlations with the brain PLFA of mice fed palm oil. Additionally, all except LDL were negatively correlated with DGLA in kidney PL in palm and linseed oil groups. LDL displayed a distinct correlation in brain PL, positively associating with ALA in the linseed oil group. Furthermore, in WAT PL, LDL was positively correlated predominantly with *n*-6 FA, known for its pro-inflammatory potential [10], after linseed and sunflower oil consumption. These findings highlight potential diet-dependent lipid interactions but require further investigation to clarify their biological significance.

While our study primarily focused on the effects of dietary fats on PLFA composition in different organs, it is important to acknowledge the broader metabolic consequences of chronic high-fat diet consumption. Recent findings indicate that a prolonged high-fat diet alters circulating bile acid profiles, impairing enterohepatic circulation and disrupting gut microbiota composition and energy metabolism [69]. Furthermore, high-fat diet-induced dysbiosis has been associated with intestinal permeability, systemic inflammation, and metabolic dysfunction, which may contribute to obesity and other chronic diseases [70]. These changes have been linked to dysregulated lipid homeostasis and an increased risk of metabolic disorders, further emphasizing the systemic effects of dietary fats beyond organ-specific phospholipid remodeling.

In this study, we focused exclusively on the distribution of fatty acids within PL without examining the specific primary PL classes. This approach may have limited our ability to fully characterize the impact of dietary fats on membrane lipid composition. Furthermore, we did not directly measure pathological consequences or functional alterations in the examined organs. Besides CRP, we did not assess other inflammatory mediators to completely interpret the complex dynamics associated with high-fat diets. Despite this, our findings provide valuable insights by demonstrating that the PLFA composition varies significantly across different organs in response to chronic high-fat diets. Specifically, we identified distinct PLFA profiles in the brain, kidneys, and white adipose tissue when subjected to diets rich in linseed, palm, or sunflower oil. These results emphasize that different dietary oils distinctly influence PLFA distribution in an organ-specific manner, contributing to our understanding of lipid metabolism and the physiological implications of dietary fat intake.

## 5. Conclusions

This study demonstrates that the PLFA profile is organ-specific and undergoes distinct alterations following the chronic consumption of linseed, palm, or sunflower oil. In the brain, the linseed oil diet led to the most notable alterations, increasing *n*-3 PUFAs, particularly ALA, which was metabolized into long-chain *n*-3 PUFAs such as EPA and DPA. A similar pattern was observed in the kidneys, while in WAT, only ALA levels were elevated, without further elongation into long-chain *n*-3 PUFAs. Palm and sunflower oils each resulted in unique PLFA distributions in the kidneys and WAT. Although palm oil is rich in PA, it did not elevate PA or total SFAs; instead, it increased LA without conversion into pro-inflammatory AA. In contrast, sunflower oil elevated PA in renal PLFA and PA and total SFAs in WAT. Nevertheless, the potential metabolic risks of increased SFAs may be counteracted by the rise in OA and total MUFAs, suggesting a complex balance of the sunflower oil effects.

Our results also revealed that chronic linseed oil consumption significantly improved the serum lipid profile. Specifically, it reduced LDL levels, increased HDL levels, and enhanced cardiovascular indices such as the atherogenic coefficient, cardiac risk ratio, and cardioprotective index, suggesting its potential to mitigate cardiovascular risk.

Our findings highlight that vegetable oils, an essential part of daily diets, significantly impact organ-specific PLFA profiles. Choosing the right dietary oils to achieve a balanced fatty acid intake is crucial for maintaining health. A combined intake of various oils may be necessary to optimize fatty acid ratios. Therefore, our future research will explore the effects of combined oil consumption on PLFA profiles in different tissues.

## Figures and Tables

**Table 1:** Brain phospholipid fatty acids composition in mice on regular chow (control group), linseed oil, palm oil, and sunflower oil diets.

Fatty Acid	Control Group (%)	Linseed Oil (%)	Palm Oil (%)	Sunflower Oil (%)
SFA	45.34 ± 1.35	42.04 ± 5.7	46.15 ± 1.15	45.64 ± 0.84
PA (16:0)	21.68 ± 0.71	21.28 ± 4.38	22.1 ± 0.56	22.31 ± 0.71
STA (18:0)	23.67 ± 0.68	20.76 ± 3.03 ^a^	24.05 ± 0.63 ^bb^	23.33 ± 0.27 ^b^
MUFA	22.91 ± 0.74	23.88 ± 4.99	22.4 ± 0.91	22.62 ± 0.73
POA (16:1, *n*-7)	0.48 ± 0.04	0.55 ± 0.12	0.44 ± 0.02	0.48 ± 0.02
OA (18:1, *n*-9)	18.46 ± 0.61	19.35 ± 3.72	18.21 ± 0.76	18.44 ± 0.61
VA (18:1, *n*-7)	3.97 ± 0.11	3.73 ± 0.76	3.75 ± 0.16	3.69 ± 0.17
PUFA	31.75 ± 1.97	32.9 ± 4.39	31.45 ± 1.87	31.74 ± 0.55
*n*-6	14.06 ± 0.54	14.7 ± 1.49	14.22± 2.5	14.55 ± 0.19
LA (18:2, *n*-6)	0.73 ± 0.04	1.29 ± 0.25 ^aaa^	1.31 ± 0.18 ^aaa^	0.89 ± 0.23 ^b,cc^
GLA (18:3, *n*-6)	0.51 ± 0.07	1.04 ± 1.38	0.51 ± 0.05	0.47 ± 0.06
DGLA (20:3, *n*-6)	0.37 ± 0.009	0.58 ± 0.09 ^aa^	0.34 ± 0.15 ^bbb^	0.36 ± 0.01 ^bb^
AA (20:4, *n*-6)	9.27 ± 0.36	6.56 ± 3.98	8.94 ± 2.64	9.84 ± 0.33
DTA (22:4, *n*-6)	3.17 ± 0.35	3.06 ± 0.94	3.12 ± 0.28	2.99 ± 0.41
*n*-3	17.69 ± 2.19	16.42 ± 3.59	17.23 ± 0.76	17.19 ± 0.39
ALA (18:3, *n*-3)	3.48 ± 2.55	1.85 ± 0.99	2.25 ± 0.23	2.16 ± 0.37
EPA (20:5, *n*-3)	0.08 ± 0.04	0.16 ± 0.03 ^aa^	0.09 ± 0.04 ^b^	0.06 ± 0.03 ^bb^
DPA (22:5, *n*-3)	0.14 ± 0.006	0.58 ± 0.1 ^aa^	0.11 ± 0.01 ^bb^	0.28 ± 0.41
DHA (22:6, *n*-3)	13.99 ± 0.42	13.82 ± 3.06	14.77 ± 0.75	14.68 ± 0.32
*n*-6/*n*-3	0.8 ± 0.09	0.89 ± 0.17	0.83 ± 0.17	0.85 ± 0.01
EPA/AA	0.008 ± 0.004	0.06 ± 0.08	0.01 ± 0.01	0.006 ± 0.003
UI	172.3 ± 4.44	161.3 ± 39.39	172.2 ± 6.11	174.5 ± 2.17
*n*-3 index	3.25 ± 0.36	3.22 ± 0.95	3.21 ± 0.28	3.05 ± 0.42

SFA—saturated fatty acid(s); PA—palmitic acid; STA—stearic acid; MUFA—monounsaturated fatty acid(s); POA—palmitoleic acid; OA—oleic acid; VA—vaccenic acid; PUFA—polyunsaturated fatty acid(s); LA—linoleic acid; GLA—gamma-linolenic acid; DGLA—dihomo-gamma-linolenic acid; AA—arachidonic acid; DTA—docosatetraenoic acid; ALA—alpha-linolenic acid; EPA—eicosapentaenoic acid; DPA—docosapentaenoic acid; DHA—docosahexaenoic acid; UI—unsaturation index. Data are given as mean ± standard deviation (SD). One-way ANOVA followed by Tukey’s post hoc test was conducted for pairwise comparisons between all groups. The statistical analysis did not include the ND values. Statistical significance is indicated as follows: ^a^ *p* < 0.05, ^aa^ *p* < 0.01, ^aaa^ *p* < 0.001 compared to the control group; ^b^ *p* < 0.05, ^bb^ *p* < 0.01, ^bbb^ *p* < 0.001 compared to linseed oil group; ^cc^ *p* < 0.01, compared to palm oil group. Sample size (n): n = 6 for all groups, except: DGLA (n = 5, palm oil group).

**Table 2:** Kidney phospholipid fatty acids composition in mice on regular chow (control group), linseed oil, palm oil, and sunflower oil diets.

Fatty Acid	Control Group (%)	Linseed Oil (%)	Palm Oil (%)	Sunflower Oil (%)
SFA	39.82 ± 3.2	33.7 ± 3.9 ^a^	33.44 ± 3.3 ^a^	37.18 ± 2.57
PA (16:0)	24.21 ± 2.17	18.23 ± 2.26 ^aaa^	17.96± 1.14 ^aaa^	25.02 ± 2.34 ^bbb,ccc^
STA (18:0)	15.61 ± 1.2	15.39 ± 2.24	15.48 ± 2.42	12.17 ± 1.67 ^a,b,c^
MUFA	23.78 ± 0.89	22.19 ± 3.11	21.01 ± 2.88	30.65 ± 7.39 ^a,b,cc^
POA (16:1, *n*-7)	2.03 ± 0.31	1.39 ± 0.28 ^a^	0.79 ± 0.23 ^aaa^	1.67 ± 0.51 ^cc^
OA (18:1, *n*-9)	19.67 ± 0.58	20.18 ± 3.12	18.91 ± 2.75	27.43 ± 7.12 ^a,b,cc^
VA (18:1, *n*-7)	2.08 ± 0.11	1.31 ± 0.36 ^aaa^	1.29 ± 0.14 ^aaa^	1.54 ± 0.26 ^aa^
PUFA	36.4 ± 3.42	44.11 ± 2.91 ^a^	45.55 ± 1.62 ^aa^	31.33 ± 5.53 ^bbb,ccc^
*n*-6	30.08 ± 2.2	28.89 ± 2.96	39.76 ± 1.6 ^aaa,bbb^	25.08 ± 3.83 ^a,ccc^
LA (18:2, *n*-6)	16.52 ± 0.99	19.18 ± 1.95	26.03 ± 3.82 ^aaa,bbb^	13.89 ± 1.23 ^bb,ccc^
GLA (18:3, *n*-6)	0.37 ± 0.07	0.46 ± 0.14	0.35 ± 0.05	0.3 ± 0.01 ^b^
DGLA (20:3, *n*-6)	0.54 ± 0.05	0.65± 0.18	0.57 ± 0.11	0.49 ± 0.12
AA (20:4, *n*-6)	11.98 ± 01.53	8.01 ± 3.29	12.14 ± 2.43	9.81 ± 3.83
DTA (22:4, *n*-6)	0.66 ± 0.06	0.54 ± 0.26	0.67 ± 0.14	0.58 ± 0.13
*n*-3	6.32 ± 1.23	15.22 ± 4.85 ^aaa^	5.78 ± 1.65 ^bbb^	5.64 ± 1.67 ^bbb^
ALA (18:3, *n*-3)	0.41 ± 0.04	5.76 ± 4.49 ^aa^	0.23 ± 0.11 ^bb^	0.27 ± 0.05 ^bb^
EPA (20:5, *n*-3)	0.08 ± 0.04	1.5 ± 0.87 ^aaa^	0.04 ± 0.07 ^bbb^	0.27 ± 0.51 ^bb^
DPA (22:5, *n*-3)	0.28 ± 0.05	1.64 ± 1.36 ^a^	0.21 ± 0.08 ^bb^	0.27 ± 0.19 ^b^
DHA (22:6, *n*-3)	5.55 ± 1.12	5.57 ± 1.22	5.30 ± 1.65	4.79 ± 1.65
*n*-6/*n*-3	4.88 ± 0.74	2.2 ± 1.16 ^a^	7.34 ± 2.05 ^a,bbb^	4.72 ± 0.78 ^b,c^
EPA/AA	0.006 ± 0.003	0.22 ± 0.12 ^aa^	0.003 ± 0.006 ^bb^	0.05 ± 0.11 ^b^
UI	146.5 ± 14.10	145.9 ± 50.45	160.8 ± 11.64	134.6 ± 20.97
*n*-3 index	5.63 ± 1.16	7.08 ± 0.93	5.33 ± 1.67	5.06 ± 1.49

SFA—saturated fatty acid(s); PA—palmitic acid; STA—stearic acid; MUFA—monounsaturated fatty acid(s); POA—palmitoleic acid; OA—oleic acid; VA—vaccenic acid; PUFA—polyunsaturated fatty acid(s); LA—linoleic acid; GLA—gamma-linolenic acid; DGLA—dihomo-gamma-linolenic acid; AA—arachidonic acid; DTA—docosatetraenoic acid; ALA—alpha-linolenic acid; EPA—eicosapentaenoic acid; DPA—docosapentaenoic acid; DHA—docosahexaenoic acid; UI—unsaturation index. Data are given as mean ± standard deviation (SD). One-way ANOVA followed by Tukey’s post hoc test was conducted for pairwise comparisons between all groups. The statistical analysis did not include the ND values. Statistical significance is indicated as follows: ^a^ *p* < 0.05, ^aa^ *p* < 0.01, ^aaa^ *p* < 0.001 compared to control group; ^b^ *p* < 0.05, ^bb^ *p* < 0.01, ^bbb^ *p* < 0.001 compared to linseed oil group; ^c^ *p* < 0.05, ^cc^ *p* < 0.01, ^ccc^ *p* < 0.001 compared to palm oil group. Sample size (n): n = 6 for all groups, except STA (n = 5, linseed oil group); GLA (n = 5, linseed oil group); DGLA (n = 5, linseed oil group); AA (n = 5, control, linseed, and sunflower oil groups).

**Table 3:** White adipose tissue phospholipid fatty acids composition in mice on regular chow (control group), linseed oil, palm oil, and sunflower oil diet.

Fatty Acid	Control Group (%)	Linseed Oil (%)	Palm Oil (%)	Sunflower Oil (%)
SFA	28.58 ± 3.51	21.6 ± 8.56	20.56 ± 6.67	31.54 ± 4.12 ^b,c^
PA (16:0)	23.03 ± 1.47	15.38 ± 4.59 ^aa^	14.75 ± 4.67 ^aa^	26.25 ± 1.88 ^bbb,ccc^
STA (18:0)	5.55 ± 2.13	6.22 ± 4.01	5.82 ± 2.15	5.28 ± 3.49
MUFA	39.68 ± 3.41	35.22 ± 3.47	36.17 ± 2.22	53.14 ± 1.31 ^aaa,bbb,ccc^
POA (16:1, *n*-7)	4.47 ± 0.79	2.54 ± 0.57 ^aaa^	1.41 ± 0.43 ^aaa^	2.82 ± 0.97 ^aa,c^
OA (18:1, *n*-9)	33.18 ± 3.29	31.42 ± 3.24	33.92 ± 1.95	44.35 ± 10.85 ^a,bb,c^
VA (18:1, *n*-7)	2.02 ± 0.14	1.27 ± 0.08 ^aaa^	0.84 ± 0.37 ^aaa,b^	1.28 ± 0.15 ^aaa,cc^
PUFA	31.84 ± 1.34	43.17 ± 5.67	48.27 ± 13.67 ^a^	16.86 ± 0.61 ^a,bbb,ccc^
*n*-6	29.98 ± 1.27	21.41 ± 1.02	47.05 ± 13.44 ^aa,bbb^	15.83 ± 0.64 ^a,ccc^
LA (18:2, *n*-6)	27.5 ± 3.8	20.25 ± 1.29	45.65 ± 13.43 ^aa,bbb^	15.36 ± 1.01 ^a,ccc^
GLA (18:3, *n*-6)	0.16 ± 0.007	0.26 ± 0.02 ^aa^	0.18 ± 0.09 ^b^	0.17 ± 0.02 ^b^
DGLA (20:3, *n*-6)	0.22 ± 0.08	0.21 ± 0.25	0.24 ± 0.05	0.18 ± 0.21
AA (20:4, *n*-6)	0.57 ± 0.04	0.41 ± 0.18	0.82 ± 0.27 ^b^	0.49 ± 0.21
DTA (22:4, *n*-6)	0.11 ± 0.06	0.17 ± 0.18	0.16 ± 0.09	0.14 ± 0.13
*n*-3	1.89 ± 0.12	21.53 ± 5.98 ^aaa^	1.21 ± 0.32 ^bbb^	1.02 ± 0.28 ^bbb^
ALA (18:3, *n*-3)	1.42 ± 0.42	20.45 ± 6.85 ^aaa^	0.97 ± 0.28 ^bbb^	0.71 ± 0.33 ^bbb^
EPA (20:5, *n*-3)	0.06 ± 0.06	0.42 ± 0.61	ND	0.11 ± 0.13
DPA (22:5, *n*-3)	0.09 ± 0.08	0.27 ± 0.17	ND	0.11 ± 0.13
DHA (22:6, *n*-3)	0.34 ± 0.32	0.39 ± 0.19	0.24 ± 0.09	0.22 ± 0.14
*n*-6/*n*-3	16.15 ± 0.82	0.89 ± 0.03 ^aa^	39.41 ± 9 ^aaa,bbb^	16.34 ± 3.93 ^bb,ccc^
EPA/AA	0.04 ± 0.006	0.44 ± 0.13 ^aa^	ND	0.009 ± 0.009 ^bb^
UI	105.0 ± 9.60	148.0 ± 15.65 ^aaa^	137.0 ± 25.67 ^a^	87.97 ± 5.98 ^bbb,ccc^
*n*-3 index	0.37 ± 0.38	0.81 ± 0.78	0.24 ± 0.09	0.50 ± 0.67

SFA—saturated fatty acid(s); PA—palmitic acid; STA—stearic acid; MUFA—monounsaturated fatty acid(s); POA—palmitoleic acid; OA—oleic acid; VA—vaccenic acid; PUFA—polyunsaturated fatty acid(s); LA—linoleic acid; GLA—gamma-linolenic acid; DGLA—dihomo-gamma-linolenic acid; AA—arachidonic acid; DTA—docosatetraenoic acid; ALA—alpha-linolenic acid; EPA—eicosapentaenoic acid; DPA—docosapentaenoic acid; DHA—docosahexaenoic acid; UI—unsaturation index; ND—not detected. Data are given as mean ± standard deviation (SD). One-way ANOVA followed by Tukey’s post hoc test was conducted for pairwise comparisons between all groups. The statistical analysis did not include the ND values. Statistical significance is indicated as follows: ^a^ *p* < 0.05, ^aa^ *p* < 0.01, ^aaa^ *p* < 0.001 compared to the control group; ^b^ *p* < 0.05, ^bb^ *p* < 0.01, ^bbb^ *p* < 0.001 compared to the linseed oil group; ^c^ *p* < 0.05, ^cc^ *p* < 0.01, ^ccc^ *p* < 0.001 compared to the palm oil group. Sample size (n): n = 6 for all groups, except OA (n = 5, sunflower oil group); GLA (n = 5, linseed oil group); DGLA (n = 5, linseed oil group); AA (n = 5, control, linseed, and sunflower oil groups); EPA (n = 5, sunflower oil group; ND, palm oil group); DPA (n = 5, sunflower oil group; ND, palm oil group); EPA/AA (n = 5, control group; n = 4, sunflower oil group; ND, palm oil group).

**Table 4:** Serum lipid profile and cardiovascular indices in mice on regular chow (control group), linseed oil, palm oil, and sunflower oil diet.

Parameters	Control Group	Linseed Oil	Palm Oil	Sunflower Oil
TC (mmol/L)	2.00 ± 0.18	2.37 ± 0.37	2.50 ± 0.54	2.45 ± 0.47
LDL (mmol/L)	0.45 ± 0.08	0.23 ± 0.13 ^a^	0.55 ± 0.16 ^bbb^	0.59 ± 0.09 ^bbb^
HDL (mmol/L)	1.03 ± 0.10	1.45 ± 0.32 ^a^	1.40 ± 0.25	1.25 ± 0.30
non-HDL-c (mmol/L)	0.96 ± 0.09	0.92 ± 0.16	1.11 ± 0.27	1.15 ± 0.20
TG (mmol/L)	1.12 ± 0.14	1.51 ± 0.29	1.23 ± 0.29	1.25 ± 0.38
AI	0.03 ± 0.05	0.02± 0.09	−0.06 ± 0.04	−0.01 ± 0.06
AC	0.93 ± 0.06	0.66 ± 0.20 ^a^	0.79 ± 0.05	0.94 ± 0.15 ^bb^
CRR	1.90 ± 0.06	1.65 ± 0.21 ^a^	1.78 ± 0.07	1.95 ± 0.14 ^bb^
CPI	2.34 ± 0.41	9.07 ± 4.93 ^aaa^	2.62 ± 0.44 ^bb^	2.16 ± 0.59 ^bbb^

TC—total cholesterol; LDL—low-density lipoprotein; HDL—high-density lipoprotein; non-HDL-c—non-HDL cholesterol; TG—triglycerides; AI—atherogenic index; AC—atherogenic coefficient; CRR—cardiac risk ratio; CPI—cardioprotective index; n = 6 for all groups. One-way ANOVA followed by Tukey’s post hoc test was conducted for pairwise comparisons between all groups. Data are given as mean ± standard deviation (SD). ^a^ *p* < 0.05; ^aaa^ *p* < 0.001 compared to the control group. ^bb^ *p* < 0.01; ^bbb^ *p* < 0.001 compared to linseed oil.

## Data Availability

The original contributions presented in the study are included in the article/Supplementary Materials, further inquiries can be directed to the corresponding authors.

## Supplementary Materials

The following supporting information can be downloaded at: https://www.mdpi.com/article/10.3390/nu17050821/s1, Supplementary Table S1. Total cholesterol (TC) correlation in mice on regular chow (control group), linseed oil, palm oil, and sunflower oil diet. Supplementary Table S2. Low-density lipoprotein (LDL) correlation in mice on regular chow (control group), linseed oil, palm oil, and sunflower oil diet. Supplementary Table S3. High-density lipoprotein (HDL) correlation in mice on regular chow (control group), linseed oil, palm oil, and sunflower oil diet. Supplementary Table S4. Triglycerides (TG) correlation in mice on regular chow (control group), linseed oil, palm oil, and sunflower oil diet.

## Abbreviations

The following abbreviations are used in this manuscript:

EFA	Essential fatty acid
LA	Linoleic acid (18:2, *n*-6)
ALA	Alpha-linolenic acid (18:3, *n*-3)
PUFA	Polyunsaturated fatty acid
EPA	Eicosapentaenoic acid (20:5, *n*-3)
DHA	Docosahexaenoic acid (22:6, *n*-3)
SFA	Saturated fatty acid
PA	Palmitic acid (16:0)
STA	Stearic acid (18:0)
MUFA	Monounsaturated fatty acid
OA	Oleic acid (18:1, *n*-9)
PL	Phospholipid
AA	Arachidonic acid (20:4, *n*-6)
PLFA	Phospholipid fatty acid
CKD	Chronic kidney disease
WAT	White adipose tissue
BHT	Butylated hydroxytoluene
TLC	Thin-layer chromatography
GLC	Gas–liquid chromatography
EA	Elongase activity
D9D	Δ9 Desaturase activity
D6D	Δ6 Desaturase activity
GLA	Gamma-linolenic acid (18:3, *n*-6)
D5D	Δ5 Desaturase activity
DGLA	Dihomo-gamma-linolenic acid (20:3, *n*-6)
TC	Total cholesterol
HDL	High-density lipoprotein
LDL	Low-density lipoprotein
TG	Triglyceride
CRP	C-reactive protein
AI	Atherogenic Index
AC	Atherogenic Coefficient
CRR	Cardiac Risk Ratio
CPI	Cardioprotective Index
non-HDL-c	non-HDL cholesterol
SD	Standard deviation
DPA	Docosapentaenoic acid (22:5, *n*-3)
POA	Palmitoleic acid (16:1, *n*-7)
VA	Vaccenic acid (18:1, *n*-7)
UI	Unsaturation index

## References

[1] Wang X., Song R., Clinchamps M., Dutheil F. (2023). New Insights into High-Fat Diet with Chronic Diseases. Nutrients.

[2] Wilson B.A., Pollard R.D., Ferguson D.S., Motarjemi Y. (2014). Nutriential Hazards: Macronutrients: Essential Fatty Acids. Encyclopedia of Food Safety.

[3] Al-Madhagy S., Ashmawy N.S., Mamdouh A., Eldahshan O.A., Farag M.A. (2023). A Comprehensive Review of the Health Benefits of Flaxseed Oil in Relation to Its Chemical Composition and Comparison with Other Omega-3-Rich Oils. Eur. J. Med. Res..

[4] Mancini A., Imperlini E., Nigro E., Montagnese C., Daniele A., Orrù S., Buono P. (2015). Biological and Nutritional Properties of Palm Oil and Palmitic Acid: Effects on Health. Molecules.

[5] Akkaya M.R. (2018). Prediction of Fatty Acid Composition of Sunflower Seeds by Near-Infrared Reflectance Spectroscopy. J. Food Sci. Technol..

[6] Ventura R., Martínez-Ruiz I., Hernández-Álvarez M.I. (2022). Phospholipid Membrane Transport and Associated Diseases. Biomedicines.

[7] Hąc-Wydro K., Wydro P. (2007). The Influence of Fatty Acids on Model Cholesterol/Phospholipid Membranes. Chem. Phys. Lipids.

[8] Wang B., Tontonoz P. (2019). Phospholipid Remodeling in Physiology and Disease. Annu. Rev. Physiol..

[9] Clemente-Suárez V.J., Beltrán-Velasco A.I., Redondo-Flórez L., Martín-Rodríguez A., Tornero-Aguilera J.F. (2023). Global Impacts of Western Diet and Its Effects on Metabolism and Health: A Narrative Review. Nutrients.

[10] Innes J.K., Calder P.C. (2018). Omega-6 Fatty Acids and Inflammation. Prostaglandins Leukot. Essent. Fat. Acids.

[11] Calder P.C. (2017). Omega-3 Fatty Acids and Inflammatory Processes: From Molecules to Man. Biochem. Soc. Trans..

[12] Gonzalez-Becerra K., Barron-Cabrera E., Muñoz-Valle J.F., Torres-Castillo N., Rivera-Valdes J.J., Rodriguez-Echevarria R., Martinez-Lopez E. (2023). A Balanced Dietary Ratio of n-6:n-3 Polyunsaturated Fatty Acids Exerts an Effect on Total Fatty Acid Profile in RBCs and Inflammatory Markers in Subjects with Obesity. Healthcare.

[13] Fenton J.I., Gurzell E.A., Davidson E.A., Harris W.S. (2016). Red Blood Cell PUFAs Reflect the Phospholipid PUFA Composition of Major Organs. Prostaglandins Leukot. Essent. Fat. Acids.

[14] Tu W.C., Mühlhäusler B.S., Yelland L.N., Gibson R.A. (2013). Correlations between Blood and Tissue Omega-3 LCPUFA Status Following Dietary ALA Intervention in Rats. Prostaglandins Leukot. Essent. Fat. Acids.

[15] Melo H.M., Santos L.E., Ferreira S.T. (2019). Diet-Derived Fatty Acids, Brain Inflammation, and Mental Health. Front. Neurosci..

[16] Emma E.M., Kiliaan, Amanda J., Custers (2022). Dietary Lipids from Body to Brain. Prog. Lipid Res..

[17] Sánchez-Alegría K., Arias C. (2022). Functional Consequences of Brain Exposure to Saturated Fatty Acids: From Energy Metabolism and Insulin Resistance to Neuronal Damage. Endocrinol. Diabetes Metab..

[18] Dighriri I.M., Alsubaie A.M., Hakami F.M., Hamithi D.M., Alshekh M.M., Khobrani F.A., Dalak F.E., Hakami A.A., Alsueaadi E.H., Alsaawi L.S. (2022). Effects of Omega-3 Polyunsaturated Fatty Acids on Brain Functions: A Systematic Review. Cureus.

[19] De Boer I.H., Utzschneider K.M. (2017). The Kidney’s Role in Systemic Metabolism—Still Much to Learn. Nephrol. Dial. Transplant..

[20] Rangel Silvares R., Nunes Goulart da Silva Pereira E., Eduardo Ilaquita Flores E., Lino Rodrigues K., Ribeiro Silva A., Gonçalves-de-Albuquerque C.F., Daliry A. (2019). High-Fat Diet-Induced Kidney Alterations in Rats with Metabolic Syndrome: Endothelial Dysfunction and Decreased Antioxidant Defense. Diabetes Metab. Syndr. Obes. Targets Ther..

[21] Sun Y., Ge X., Li X., He J., Wei X., Du J., Sun J., Li X., Xun Z., Liu W. (2020). High-Fat Diet Promotes Renal Injury by Inducing Oxidative Stress and Mitochondrial Dysfunction. Cell Death Dis..

[22] Kawai T., Autieri M.V., Scalia R. (2021). Adipose Tissue Inflammation and Metabolic Dysfunction in Obesity. Am. J. Physiol.-Cell Physiol..

[23] Piché M.-E., Tchernof A., Després J.-P. (2020). Obesity Phenotypes, Diabetes, and Cardiovascular Diseases. Circ. Res..

[24] Kahn C.R., Wang G., Lee K.Y. (2019). Altered Adipose Tissue and Adipocyte Function in the Pathogenesis of Metabolic Syndrome. J. Clin. Investig..

[25] Popović T., Nenadović A., Stanković A., Debeljak Martačić J., Ranković S., Kovačević S., Nešović Ostojić J., Ilić A., Milašin J., De Luka S. (2024). Liver Phospholipid Fatty Acid Composition in Response to Chronic High-Fat Diets. Biochim. Biophys. Acta (BBA)-Mol. Cell Biol. Lipids.

[26] Schreyer S.A., Wilson D.L., LeBoeuf R.C. (1998). C57BL/6 Mice Fed High Fat Diets as Models for Diabetes-Accelerated Atherosclerosis. Atherosclerosis.

[27] Funkat A., Massa C.M., Jovanovska V., Proietto J., Andrikopoulos S. (2004). Metabolic Adaptations of Three Inbred Strains of Mice (C57BL/6, DBA/2, and 129T2) in Response to a High-Fat Diet. J. Nutr..

[28] Dutta S., Sengupta P. (2016). Men and Mice: Relating Their Ages. Life Sci..

[29] Arifin W.N., Zahiruddin W.M. (2017). Sample Size Calculation in Animal Studies Using Resource Equation Approach. Malays. J. Med. Sci..

[30] Folch J., Lees M., Sloane Stanley G.H. (1957). A Simple Method for the Isolation and Purification of Total Lipids from Animal Tissues. J. Biol. Chem..

[31] Daneshmand R., Kurl S., Tuomainen T.-P., Virtanen J.K. (2017). Associations of Estimated Δ-5-Desaturase and Δ-6-Desaturase Activities with Stroke Risk Factors and Risk of Stroke: The Kuopio Ischaemic Heart Disease Risk Factor Study. Br. J. Nutr..

[32] Domínguez-López I., Arancibia-Riveros C., Tresserra-Rimbau A., Castro-Barquero S., Casas R., Vázquez-Ruiz Z., Ros E., Fitó M., Estruch R., López-Sabater M.C. (2022). Relationship between Estimated Desaturase Enzyme Activity and Metabolic Syndrome in a Longitudinal Study. Front. Nutr..

[33] Oršolić N., Landeka Jurčević I., Đikić D., Rogić D., Odeh D., Balta V., Perak Junaković E., Terzić S., Jutrić D. (2019). Effect of Propolis on Diet-Induced Hyperlipidemia and Atherogenic Indices in Mice. Antioxidants.

[34] Gimenez M.S., Oliveros L.B., Gomez N.N. (2011). Nutritional Deficiencies and Phospholipid Metabolism. Int. J. Mol. Sci..

[35] Bacle A., Kadri L., Khoury S., Ferru-Clément R., Faivre J.-F., Cognard C., Bescond J., Krzesiak A., Contzler H., Delpech N. (2020). A Comprehensive Study of Phospholipid Fatty Acid Rearrangements in Metabolic Syndrome: Correlations with Organ Dysfunction. Dis. Models Mech..

[36] Paiva K.M., Oliveira R.F., Helena L., Madruga A., Sousa G., Freire K.F., De J., Araújo L., Lopes R. (2023). Physical Exercise and Flaxseed Oil Supplementation Influence the Glial Plasticity in the Rat Hippocampus. Acta Neurobiol. Exp..

[37] Butler M.J., Mackey-Alfonso S.E., Massa N., Baskin K.K., Barrientos R.M. (2023). Dietary Fatty Acids Differentially Impact Phagocytosis, Inflammatory Gene Expression, and Mitochondrial Respiration in Microglial and Neuronal Cell Models. Front. Cell. Neurosci..

[38] Wysoczański T., Sokoła-Wysoczańska E., Pękala J., Lochyński S., Czyż K., Bodkowski R., Herbinger G., Patkowska-Sokoła B., Librowski T. (2016). Omega-3 Fatty Acids and Their Role in Central Nervous System—A Review. Curr. Med. Chem..

[39] Fisette A., Sergi D., Breton-Morin A., Descôteaux S., Martinoli M.-G. (2022). New Insights on the Role of Bioactive Food Derivatives in Neurodegeneration and Neuroprotection. Curr. Pharm. Des..

[40] Liu B., Zhang Y., Yang Z., Liu M., Zhang C., Zhao Y., Song C. (2021). ω-3 DPA Protected Neurons from Neuroinflammation by Balancing Microglia M1/M2 Polarizations through Inhibiting NF-ΚB/MAPK P38 Signaling and Activating Neuron-BDNF-PI3K/AKT Pathways. Mar. Drugs.

[41] Song J., Kim Y.-S., Lee D.H., Lee S.H., Park H.J., Lee D., Kim H. (2019). Neuroprotective Effects of Oleic Acid in Rodent Models of Cerebral Ischaemia. Sci. Rep..

[42] Wang X., Lin H., Gu Y. (2012). Multiple Roles of Dihomo-γ-Linolenic Acid against Proliferation Diseases. Lipids Health Dis..

[43] Mustonen A.-M., Nieminen P. (2023). Dihomo-γ-Linolenic Acid (20:3n-6)—Metabolism, Derivatives, and Potential Significance in Chronic Inflammation. Int. J. Mol. Sci..

[44] Chang Z., Yang M., Ji H. (2021). Molecular Characterization and Functional Analysis of Apoptosis-Inducing Factor (AIF) in Palmitic Acid-Induced Apoptosis in Ctenopharyngodon Idellus Kidney (CIK) Cells. Fish Physiol. Biochem..

[45] Gillingham L.G., Harris-Janz S., Jones P.J.H. (2011). Dietary Monounsaturated Fatty Acids Are Protective against Metabolic Syndrome and Cardiovascular Disease Risk Factors. Lipids.

[46] Gwon D., Hwang T., Ro J.-Y., Kang Y.-J., Jeong J., Kim D.-K., Lim K., Kim D., Choi D., Kim J.-J. (2017). High Endogenous Accumulation of ω-3 Polyunsaturated Fatty Acids Protect against Ischemia-Reperfusion Renal Injury through AMPK-Mediated Autophagy in Fat-1 Mice. Int. J. Mol. Sci..

[47] Ajami M., Davoodi S.H., Habibey R., Namazi N., Soleimani M., Pazoki-Toroudi H. (2012). Effect of DHA+EPA on Oxidative Stress and Apoptosis Induced by Ischemia-Reperfusion in Rat Kidneys. Fundam. Clin. Pharmacol..

[48] Ashtiyani S.C., Najafi H., Kabirinia K., Vahedi E., Jamebozorky L. (2012). Oral Omega-3 Fatty Acid for Reduction of Kidney Dysfunction Induced by Reperfusion Injury in Rats. Iran. J. Kidney Dis..

[49] Liput K.P., Lepczyński A., Ogłuszka M., Nawrocka A., Poławska E., Grzesiak A., Ślaska B., Pareek C.S., Czarnik U., Pierzchała M. (2021). Effects of Dietary N–3 and N–6 Polyunsaturated Fatty Acids in Inflammation and Cancerogenesis. Int. J. Mol. Sci..

[50] Shioda R., Jo-Watanabe A., Lee-Okada H.-C., Yasukawa K., Okuno T., Suzuki Y., Yokomizo T. (2021). Dietary Intake of N-3 Polyunsaturated Fatty Acids Alters the Lipid Mediator Profile of the Kidney but Does Not Attenuate Renal Insufficiency. Biochem. Biophys. Res. Commun..

[51] Rund K.M., Peng S., Greite R., Claaßen C., Nolte F., Oger C., Galano J.-M., Balas L., Durand T., Chen R. (2019). Dietary Omega-3 PUFA Improved Tubular Function after Ischemia Induced Acute Kidney Injury in Mice but Did Not Attenuate Impairment of Renal Function. Prostaglandins Other Lipid Mediat..

[52] Liu K., Acharjee A., Hinz C., Liggi S., Murgia A., Denes J., Gulston M.K., Wang X., Chu Y., West J.A. (2020). Consequences of Lipid Remodeling of Adipocyte Membranes Being Functionally Distinct from Lipid Storage in Obesity. J. Proteome Res..

[53] Holzer R.G., Park E.-J., Li N., Tran H., Chen M., Choi C., Solinas G., Karin M. (2011). Saturated Fatty Acids Induce C-Src Clustering within Membrane Subdomains, Leading to JNK Activation. Cell.

[54] Solinas G., Naugler W., Galimi F., Lee M.-S., Karin M. (2006). Saturated Fatty Acids Inhibit Induction of Insulin Gene Transcription by JNK-Mediated Phosphorylation of Insulin-Receptor Substrates. Proc. Natl. Acad. Sci. USA.

[55] Palmgren H., Petkevicius K., Bartesaghi S., Ahnmark A., Ruiz M., Nilsson R., Löfgren L., Glover M.S., Andréasson A.-C., Andersson L. (2022). Elevated Adipocyte Membrane Phospholipid Saturation Does Not Compromise Insulin Signaling. Diabetes.

[56] Ravaut G., Légiot A., Bergeron K.-F., Mounier C. (2020). Monounsaturated Fatty Acids in Obesity-Related Inflammation. Int. J. Mol. Sci..

[57] Ralston J.C., Metherel A.H., Stark K.D., Mutch D.M. (2016). SCD1 Mediates the Influence of Exogenous Saturated and Monounsaturated Fatty Acids in Adipocytes: Effects on Cellular Stress, Inflammatory Markers and Fatty Acid Elongation. J. Nutr. Biochem..

[58] Martins B.C., Ribeiro S., Teixeira S., Peixoto T.C., Lisboa P.C., Martins F.F., Souza-Mello V., Daleprane J.B. (2024). Consumption of Interesterified Palm Oil Leads Inflammation of White Adipose Tissue and Triggers Metabolic Disturbances in Mice on a High-Fat Diet. Sci. Rep..

[59] Li R., Cao C., Zheng Z., Yang X., Tan C.P., Xu Y., Liu Y. (2021). Palm Oil Consumption and Its Repercussion on Endogenous Fatty Acids Distribution. Food Funct..

[60] Saavedra-Peña R.D.M., Taylor N., Flannery C., Rodeheffer M.S. (2023). Estradiol Cycling Drives Female Obesogenic Adipocyte Hyperplasia. Cell Rep..

[61] Tóth M.E., Dukay B., Péter M., Balogh G., Szűcs G., Zvara Á., Szebeni G.J., Hajdu P., Sárközy M., Puskás L.G. (2021). Male and Female Animals Respond Differently to High-Fat Diet and Regular Exercise Training in a Mouse Model of Hyperlipidemia. Int. J. Mol. Sci..

[62] Walker C.G., Browning L.M., Mander A.P., Madden J., West A.L., Calder P.C., Jebb S.A. (2013). Age and Sex Differences in the Incorporation of EPA and DHA into Plasma Fractions, Cells and Adipose Tissue in Humans. Br. J. Nutr..

[63] Seike M., Ashida H., Yamashita Y. (2023). Dietary Flaxseed Oil Induces Production of Adiponectin in Visceral Fat and Prevents Obesity in Mice. Nutr. Res..

[64] Baranowski M., Enns J., Blewett H., Yakandawala U., Zahradka P., Taylor C.G. (2012). Dietary Flaxseed Oil Reduces Adipocyte Size, Adipose Monocyte Chemoattractant Protein-1 Levels and T-Cell Infiltration in Obese, Insulin-Resistant Rats. Cytokine.

[65] Rodriguez D., Lavie C.J., Elagizi A., Milani R.V. (2022). Update on Omega-3 Polyunsaturated Fatty Acids on Cardiovascular Health. Nutrients.

[66] Parikh M., Pierce G.N. (2019). Dietary Flaxseed: What We Know and Don’t Know about Its Effects on Cardiovascular Disease. Can. J. Physiol. Pharmacol..

[67] Dupasquier C.M.C., Dibrov E., Kneesh A.L., Cheung P.K.M., Lee K.G.Y., Alexander H.K., Yeganeh B.K., Moghadasian M.H., Pierce G.N. (2007). Dietary Flaxseed Inhibits Atherosclerosis in the LDL Receptor-Deficient Mouse in Part through Antiproliferative and Anti-Inflammatory Actions. Am. J. Physiol.-Heart Circ. Physiol..

[68] Ismail S.R., Maarof S.K., Siedar A.S., Ali A. (2018). Systematic Review of Palm Oil Consumption and the Risk of Cardiovascular Disease. PLoS ONE.

[69] Cai H., Zhang J., Liu C., Le T.N., Lu Y., Feng F., Zhao M. (2024). High-Fat Diet-Induced Decreased Circulating Bile Acids Contribute to Obesity Associated with Gut Microbiota in Mice. Foods.

[70] Malesza I.J., Malesza M., Walkowiak J., Mussin N., Walkowiak D., Aringazina R., Bartkowiak-Wieczorek J., Mądry E. (2021). High-Fat, Western-Style Diet, Systemic Inflammation, and Gut Microbiota: A Narrative Review. Cells.

