# Supplementary material for: Organ-Specific Responses to Chronic High-Fat Diets in Mice: Insights into Phospholipid Fatty Acid Distribution
Source: Nutrients. 2025 Feb 27;17(5):821. doi: 10.3390/nu17050821 (PMC11901986; doi:10.3390/nu17050821)
Supplement: Supplementary file 1 [file nutrients-17-00821-s001.zip › nutrients-3488825-supplementary.pdf]

Supplementary Table S1. Total cholesterol (TC) correlation in mice on regular chow (control group), linseed oil, palm oil, and sunflower oil diet.

Variable	r	p
	**BRAIN**	
	Palm oil	
SFA	-0.9292	0.0073
PA (16:0)	-0.8400	0.0364
LA (18:2, n-6)	-0.8452	0.0341
n3	-0.9329	0.0066
DHA (22:6, n-3)	-0.8644	0.0263
EPA/AA	-0.8948	0.0160
	**KIDNEY**	
	Linseed oil	
DGLA (20:3, n-6)	-0.8786	0.0499
DPA (22:5, n-3)	-0.9373	0.0058
	Palm oil	
DGLA (20:3, n-6)	-0.8321	0.0399

SFA – saturated fatty acid(s); PA – palmitic acid; LA – linoleic acid; DHA - docosahexaenoic acid; EPA – eicosapentaenoic acid; AA – arachidonic acid; DGLA – dihomo-gamma-linolenic acid; DPA – docosapentaenoic acid.

Pearson correlation was used for variables with homogeneous distributions, while Spearman's rank correlation was applied for non-homogeneous distributions. The tables display correlation coefficients (r) and corresponding p-values for each variable. Negative correlation coefficients indicate an inverse relationship between the variables, while positive values indicate a direct relationship. Only statistically significant correlations are presented. Statistical significance was considered relevant for $p < 0.05$.

Sample size (n): n=6 for all groups, except: DGLA (n=5, linseed oil group in kidney).

Supplementary Table S2. Low-density lipoprotein (LDL) correlation in mice on regular chow (control group), linseed oil, palm oil, and sunflower oil diet.

Variable	r	p
	**BRAIN**	
	Linseed oil	
ALA (18:3, n-3)	0.8860	0.0188
	Palm oil	
SFA	-0.8119	0.0498
	**WHITE ADIPOSE TISSUE**	
	Linseed oil	
n6	0.9041	0.0352
EPA/AA	0.8418	0.0355
	Sunflower oil	
n-6	0.9068	0.0337
AA (20:4, n-6)	0.9645	0.0080

DHA (22:6, n-3)	0.9007	0.0370
UI	0.8383	0.0371

ALA – alpha-linolenic acid; SFA – saturated fatty acid(s); EPA – eicosapentaenoic acid; AA – arachidonic acid; DHA - docosahexaenoic acid; UI – unsaturation index.

Pearson correlation was used for variables with homogeneous distributions, while Spearman's rank correlation was applied for non-homogeneous distributions. The tables display correlation coefficients (r) and corresponding p-values for each variable. Negative correlation coefficients indicate an inverse relationship between the variables, while positive values indicate a direct relationship. Only statistically significant correlations are presented. Statistical significance was considered relevant for $p < 0.05$.

Sample size (n): n=6 for all groups, except: AA (n=5, sunflower oil group in white adipose tissue).

Supplementary Table S3. High-density lipoprotein (HDL) correlation in mice on regular chow (control group), linseed oil, palm oil, and sunflower oil diet.

Variable	r	p
	**BRAIN**	
	Palm oil	
SFA	-0.8990	0.0148
LA (18:2, n-6)	-0.8683	0.0249
n3	-0.9682	0.0015
DHA (22:6, n-3)	-0.8978	0.0151
EPA/AA	-0.9394	0.0054
	**KIDNEY**	
	Linseed oil	
LA (18:2, n-6)	-0.8912	0.0424
DHA (22:6, n-3)	0.8795	0.0493
	Palm oil	
DGLA (20:3, n-6)	-0.8819	0.0201

SFA – saturated fatty acid(s); LA – linoleic acid; DHA - docosahexaenoic acid; EPA – eicosapentaenoic acid; AA – arachidonic acid; DGLA – dihomo-gamma-linolenic acid.

Pearson correlation was used for variables with homogeneous distributions, while Spearman's rank correlation was applied for non-homogeneous distributions. The tables display correlation coefficients (r) and corresponding p-values for each variable. Negative correlation coefficients indicate an inverse relationship between the variables, while positive values indicate a direct relationship. Only statistically significant correlations are presented. Statistical significance was considered relevant for $p < 0.05$.

Sample size (n): n=6 for all groups.

Supplementary Table S4. Triglycerides (TG) correlation in mice on regular chow (control group), linseed oil, palm oil, and sunflower oil diet.

Variable	r	p
	**BRAIN**	
	Palm oil	
SFA	-0.9194	0.0095
PA (16:0)	-0.8884	0.0180

POA (16:1, n-7)	-0.8846	0.0192
n-3	-0.8947	0.0160
DHA (22:6, n-3)	-0.8829	0.0198
**KIDNEY**		
Linseed oil		
DGLA (20:3, n-6)	-0.8866	0.0450

SFA – saturated fatty acid(s); PA – palmitic acid; POA – palmitoleic acid; DHA - docosahexaenoic acid; DGLA – dihomo-gamma-linolenic acid.

Pearson correlation was used for variables with homogeneous distributions, while Spearman's rank correlation was applied for non-homogeneous distributions. The tables display correlation coefficients (r) and corresponding p-values for each variable. Negative correlation coefficients indicate an inverse relationship between the variables, while positive values indicate a direct relationship. Only statistically significant correlations are presented. Statistical significance was considered relevant for $p < 0.05$.

Sample size (n): n=6 for all groups, except: DGLA (n=5, linseed oil group in kidney).
